# Targeting SYK of monocyte-derived macrophages regulates liver fibrosis via crosstalking with Erk/Hif1α and remodeling liver inflammatory environment

**DOI:** 10.1038/s41419-021-04403-2

**Published:** 2021-12-01

**Authors:** Xuejiao Chen, Ziyi Wang, Sheng Han, Zeng Wang, Yu Zhang, Xiangdong Li, Nan Xia, Wenjie Yu, Chenyang Jia, Yong Ni, Liyong Pu

**Affiliations:** 1 Hepatobiliary Center, The First Affiliated Hospital of Nanjing Medical University, Nanjing, China; 2 Key Laboratory of Liver Transplantation, Chinese Academy of Medical Sciences, Nanjing, China; 3 NHC Key Laboratory of Living Donor Liver Transplantation (Nanjing Medical University), Nanjing, Jiangsu Province China; 4 Department of Hepatopancreatobiliary Surgery, Shenzhen Second People’s Hospital, The First Affiliated Hospital of Shenzhen University, Shenzhen, Guangdong China

**Keywords:** Mechanisms of disease, Monocytes and macrophages

## Abstract

Liver fibrosis is a danger signal indicating a huge risk of liver cancer occurrence, but there is still no effective clinical means to regulate the progress of liver fibrosis. Although a variety of drugs targeting SYK have been developed for tumors and autoimmune diseases, the mechanism and specific efficacy of SYK’s role in liver fibrosis are not yet clear. Our studies based on chronic CCL4, bile duct ligation, and subacute TAA mouse models show that SYK in monocyte-derived macrophages (MoMFs) is fully dependent on phosphorylation of Erk to up-regulate the expression of Hif1α, thereby forming the crosstalk with SYK to drive liver fibrosis progress. We have evaluated the ability of the small molecule SYK inhibitor GS9973 in a variety of models. Contrary to previous impressions, high-frequency administration of GS9973 will aggravate CCL4-induced liver fibrosis, which is especially unsuitable for patients with cholestasis whose clinical features are bile duct obstruction. In addition, we found that inhibition of MoMFs SYK impairs the expression of CXCL1, on one hand, it reduces the recruitment of CD11bhiLy6Chi inflammatory cells, and on the other hand, it promotes the phenotype cross-dress process of pro-resolution MoMFs, thereby remodeling the chronic inflammatory environment of the fibrotic liver. Our further findings indicate that on the basis of the administration of CCR2/CCR5 dual inhibitor Cenicriviroc, further inhibiting MoMFs SYK may give patients with fibrosis additional benefits.

## Introduction

Liver fibrosis is a chronic liver disease characterized by chronic tissue damage and the accumulation of extracellular protein matrix [1]. The main causes of liver fibrosis are viral hepatitis, alcoholic liver disease, and non-alcoholic fatty liver. Repeated chronic damage to the liver stimulates hepatic stellate cells (HSC) to synthesize and accumulate excessive extracellular matrix [2]. Liver fibrosis progresses to liver cirrhosis and thus becomes an important risk factor for the progression of liver cancer. The diagnosis methods of liver fibrosis include invasive and non-invasive, but pathological biopsy is still the gold standard. Ishak score (Ishak0–6) is commonly used clinically to classify the severity of liver fibrosis [3].

Spleen tyrosine kinase (SYK) is a 72 kDa non-receptor tyrosine kinase that plays a multiple roles in human immune response [4]. Existing studies have shown that SYK has expressed in other tissue cells [5] and plays a wide range of physiological and pathological functions [6], which is suitable for the liver [5]. Studies have shown that the use of SYK kinase inhibitors to block SYK phosphorylation can effectively reduce the development of various liver diseases, including liver fibrosis, alcoholic liver disease, non-alcoholic fatty liver, hepatocellular carcinoma [5, 7]. Antagonistic SYK can inhibit liver fibrosis by inhibiting HSC activation, and the expression of SYK reflects the inducing stage of fibrosis to a certain extent. It is already known that the myeloid SYK inhibitor (Piceatannol, PRT06260) can effectively reduce liver fibrosis, hepatocyte damage and inflammation. GS9973 is a new type, selective SYK inhibitor has been shown to have anti-fibrosis activity in vivo [8].

The Erk signaling pathway is one of the keys to regulate the main phenotypic response of fibroblasts. Erk1/2 may be activated by MERTK from the macrophages surface, and TGF-β transcription factor regulatory protein-1 (AP-1) can be activated by Erk1/2, in addition, Erk1/2 can activate TGFβ1 through the GAS pathway [9]. The Erk pathway acts actively role in driving liver fibrosis by targeting HSC [10].

HiF1α is closely related to the development of hepatic fibrosis, and liver fibrosis caused by sinus morphological imbalances and functional blocks cause hepatocytes to hypoxia, which in turn causes degradation of HIF1α to be inhibited, thereby increasing expressions [11]. HiF1α lacks fibrosis of mice liver that relieves biliary tube ligation [12]. The expression level of liver α-SMA and COL1A1/2 of BDL mice after myeloid knockout HiF1α or HIF1β is significantly reduced compared with WT mice [13]. In addition, HIF1α is detected in liver macrophages in patients with cirrhosis. Erk1/2 has proven to be important signals for regulating HIF1α. Over-expression Erk1 significantly enhanced the activity of HIF1α under hypoxia [14], HIF1α and Erk expression increased with CCL4-induced rat liver fibrosis [15].

Despite the effectiveness of targeting myeloid SYK in mouse fibrosis, our research highlights the effectiveness of SYK in mono-derived macrophages. On one aspect, Knocked down the SYK in MoMFs impairs the expression of CXCL1 and increases the raising of LY6Chi inflammatory cells. On the other aspect, SYK crossed with the Erk/HIF1α axis and plays a crucial effect in the progress of driving fibrosis. In addition to clarifying the possible mechanism of SYK driving liver fibrosis in MoMFs, we also evaluated the effectiveness of GS9973, and the results were not ideal. It is worth noting that we have proved that target MoMFs SYK to enhance CCR2/CCR5 dual inhibitors Cernicriviroc inhibit the progress of fibrosis.

## Methods and materials

### Patient samples

Collected liver samples from patients undergoing liver resection at the Hepatobiliary Center of the First Affiliated Hospital of Nanjing Medical University, and collected and analyzed patient samples at different stages of liver fibrosis according to the Ishak classification. All patients who donated samples for this project signed informed consent, and all experiments were approved by the Ethics Committee of Nanjing Medical University.

### Murine liver fibrosis models

CCL4 induced mouse fibrosis model: 6–8 weeks old C57/BL6 male mice purchased from Vital River Laboratory Animal Technology Co. Ltd (Beijing, China) were injected intraperitoneally with CCL4 (0.6 ul/g body weight) (CCL4: olive oil = 1:3) every 3 days, continuously administered for 4–8 weeks. Inhibitor GS9973 (20 mg/kg) was dissolved in DMSO, prepared as a working solution with olive oil, and injected every 2 days. One day after the last administration of CCL4, the mice were sacrificed and the liver and serum were harvested.

Bile duct ligation (BDL) model: Adult mice were anesthetized with isoflurane inhalation. After opening the abdominal cavity, the hilar of the liver was exposed, and the common bile duct was ligated with 4–0 vicryl rapide. The sham operation group received the same procedure except for the ligation of the common bile duct. The mice were sacrificed 2–4 weeks after the operation and the liver and serum were obtained (at least 5 mice/group). All mice were numbered by one experimenter and treated blindly or operated on by another experimenter.

### TAA-induced liver fibrosis models

6–8 weeks wild-type C57/BL6 male mice were given a single sublethal dose (100 mg/kg, ip) injection of hepatotoxin thioacetamide(TAA) intraperitoneally. The mice were sacrificed 1d, 3d, and 7d after injection, and livers were obtained simultaneously. For the siSYK-mannosepolymer treatment group, mice were injected into the tail vein 4 h before TAA injection.

### Knockdown of SYK in vivo/vitro

In vivo: The SYK siRNA (20 mg/kg, Santa Cruze, California, USA) was coupled with a mannose-conjugated polymer (polyplus transfection, Illkich, France), and injected into the tail vein of mice (200 mg/kg, twice) 4 h before the induction of inflammation to Delivery to liver CD206hi macrophages.

In vitro: The shSYK lentivirus (Genepharm, Shanghai, China) was synthesized to transfect BMDMs collected from WT B6 mice. BMDM was transfected 1 day before stimulating with LPS.

### Isolation and culture of liver macrophages

Preheated PBS was used to perfuse the liver (5 ml/min). After the liver became completely white, the liver was perfused with type IV collagenase (Sigma Aldrich, 2 ml/min) dissolved in HBSS. After removing the gallbladder, the liver was harvested, and the digested cells were filtered with a 70 um cell strainer (Fisherbrand) to prepare a single-cell suspension. After centrifugation, discard the hepatic parenchymal cells, add 50% and 25% Percoll, centrifuge at 890 g for 15 min, aspirate the cells in the middle layer and continue to culture after centrifugation to obtain adherent macrophages.

### Extraction of bone marrow-derived macrophages (BMDM)

Bone marrow cells were isolated from the femur and tibia of mice. Filter through a 70 um filter and use red blood cell lysate to remove red blood cells. Cells were then cultured in DMEM supplemented with 10% FBS and 20% L929-conditioned medium, and the culture medium was replaced after 7 days for subsequent experiments.

### Western blot

After lysing the tissues and cells with RIPA, add the loading buffer water bath. After SDS-PAGE gel electrophoresis, transfer to PVDF membrane. Use for SYK (Cell Signaling Technology, 13198), p-SYK (Bioss, bs-3434R), Erk1 + Erk2 (abcam, ab184699), p-Erk1 + p-Erk2 (abcam, ab214036), HIF1α (Cell Signaling Technology, 36169), TGF-β (abcam, ab215715), α-SMA (Cell Signaling Technology, 19245), β-actin (Cell Signaling Technology, 4970) antibody for detection, using chemiluminescence-based method for color development.

### qPCR

After extracting total RNA from tissues or cells, it is reverse transcribed into cDNA. SYBR Green fluorescent dye was used for real-time fluorescence quantitative polymerase chain reaction, all expression level and results of the target gene are standardized for the GAPDH expression.

### Determination of serum ALT, AST

The measured biochemical markers of liver function included alanine aminotransferase (ALT), aspartate aminotransferase (AST). Whole blood samples were collected from mice’s intravalains placed at 4 °C overnight, and then 3000 rpm for 15 min. Using the corresponding kit (S03030, S03040) to export the results after the automatic biochemical analyzer (Rayto Life and Analytical Sciences Co., Ltd. Chemray 240).

### Immunohistochemistry and immunofluorescence

#### IHC

Fix liver tissue samples in 4% paraformaldehyde, embed in paraffin, slice into 4um-thick sections and then perform (hematoxylin-eosin) H & E staining, Sirius red staining, and masson staining. Refer to the ishak scoring system for blind evaluation of fibrosis samples.

#### Immunofluorescence

The fixed tissue sections are placed in EDTA antigen retrieval buffer (PH = 9.0), and then boiled in the microwave for repair. Block BSA for 30 min, add the corresponding primary antibodies: p-SYK(Bioss, bs-3434R), p-Erk1 + p-Erk2(abcam, ab214036), α-SMA(Cell Signaling Technology,19245), and incubate overnight. After washing with PBS, add the secondary antibody of the corresponding species. After incubating in the dark for 50 min, nuclei were stained. observe the slides under a confocal fluorescence microscope (NIKON ECLIPSE C1) and images were collected.

### Flow cytometry

The obtained macrophage lysed red blood cells were added to cell staining buffer (biolegend, 420201) to make a single-cell suspension, and Fc receptor blocker was added to reduce non-specific staining. For mouse CD11b(Biolegend,101206) and Ly6C(Biolegend,128012), F4/80(Biolegend,123110), they were processed with a flow cytometer (Beckman, Gallios) after incubation. The data were analyzed with Flow Jo.

### Statistical analysis

The data are expressed as mean ± SD, and the statistical difference between subgroups is determined by GraphPad Prism software. Student’s *t* test (and Nonparametric test), one-way ANOVA were used to compare the differences of each group. A P value less than 0.05 is considered statistically significant. One experimenter numbered patient and mouse liver tissue before the experiment and randomly assigned it to another experimenter for blind evaluation. Results were analyzed by at least two other qualified experimenters.

## Results

### P-SYK contributes to the progression of liver fibrosis in humans and mice

**Fig. 1:**
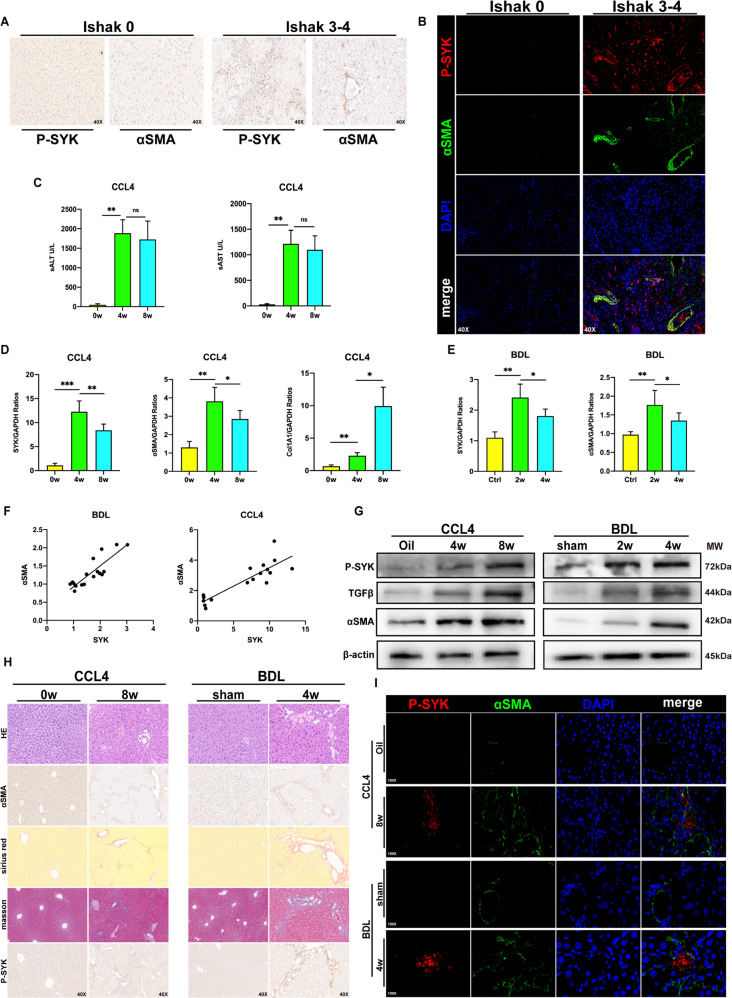
p-SYK is essential for human and mice liver fibrosis. **A** Immunohistochemical staining of p-SYK and α-SMA in liver tissues of fibrosis patients with different ishak grades (Ishak0, Ishak3–4) (magnification 40X). **B** Confocal immunofluorescence staining of p-SYK and α-SMA in liver tissues of fibrosis patients with different ishak grades (Ishak0, Ishak3–4) (magnification 40X), DAPI as nuclear localization. **C** Serum ALT and AST levels in mice injected with CCL4 for 4 and 8 weeks, oil as a control. **D** Assessed the expression of SYK, α-SMA, and col1A1 in the liver of mice treated with CCL4 for 4 and 8 weeks were by qPCR, and oil was used as a control. **E** The expression of SYK and α-SMA in the liver of mice 2 and 4 weeks after BDL surgery were determined by qPCR, and sham was used as a control. **F** Correlation analysis between the expression of SYK and α-SMA in CCL4 and BDL models. **G** Western Blot was used to analyze the protein level of p-SYK, TGF-β and α-SMA in the liver of mice injected with CCL4 for 4 or 8 weeks and mice 2 or 4 weeks after BDL, oil and sham were used as controls, respectively. **H** HE, Sirius red, masson staining and immunohistochemistry of α-SMA and p-SYK were performed on the livers of mice given CCL4 for 8 weeks and mice 4 weeks after BDL, respectively. Oil and sham were used as controls. **I** Confocal immunofluorescence staining of p-SYK and α-SMA were performed on the livers of a mouse injected with CCL4 for 8 weeks and mice 4 weeks after BDL, respectively, with DAPI as the nuclear localization.

P-SYK based on the background of NASH has been found to exert multiple effects on aggravating liver tissue damage and inflammation, and promoting the development of liver steatosis [16]. In order to ask whether the phosphorylated SYK is involved in the progress of liver fibrosis, we collected fibrotic liver tissues of patients with Ishak classification as the clinicopathological standard and found that p-SYK expressed in different Ishak Graded fibrotic livers were all up-regulated and positively correlated with the severity of fibrosis (Fig. 1A). The localization of p-SYK and α-SMA in the liver tissue of the patient was examined by confocal fluorescence microscopy. p-SYK was significantly concentrated in the area of collagen deposition, which suggested the driving effect of p-SYK on fibrosis (Fig. 1B). Subsequently, we constructed a CCL4-induced liver fibrosis model in mice and checked the liver injury levels at 4 and 8 weeks after induction. The transcription levels of SYK, α-SMA, and col1A1 were up-regulated in the inflammatory fibrotic liver with the high level of ALT and AST (Fig. 1C, D), and consistent results were also obtained in mouse liver tissues 2 and 4 weeks after BDL (Fig. 1E). In addition, the transcription level of SYK is positively correlated with αSMA. It is well known that α-SMA is usually used to assess the degree of fibrosis (Fig. 1F). In response to the progression of liver fibrosis, the protein expression of SYK and p-SYK also faithfully reflects the trend of changes in transcription levels, although the changes in activated phosphorylated SYK are more significant (Fig. 1G). In fibrotic livers of mice, αSMA and p-SYK expression were increased(Fig. 1H). The co-location of p-SYK and αSMA in fibrotic livers were increased compared with the control group and sham group (Fig. 1I). Our results prove that p-SYK plays an important role in the process of liver fibrosis. The conclusions of liver slices from the mouse fibrosis model were similar to humans.

### Blocking MoMFs SYK inhibits liver fibrosis

**Fig. 2:**
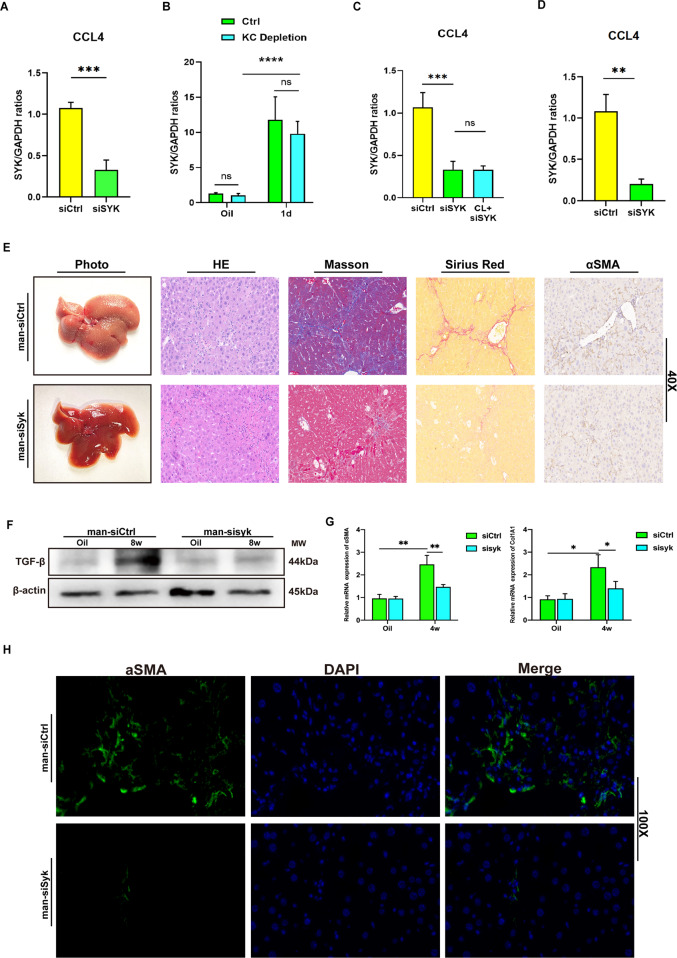
Blocking MoMFs SYK helped relieve mice liver fibrosis. **A** C57 mice were treated with a mixture of siSYK or siCtrl and mannose-conjugated JetPEI-polyplus (mannose-conjugated, JetPEI-polyplus) 4 h before the injection of CCL4 through the tail vein to blockade the transcription of SYK in liver macrophages. The mRNA level of liver SYK in siSYK or siCtrl groups was detected by qPCR 24 h after the injection of CCL4 (***P < 0.001); **B** Depleted Kupffer cells with CL before CCL4 injection, then detected SYK mRNA levels 24 h later (****P < 0.0001); **C** The level of SYK mRNA of siCtrl, siSYK and CL + siSYK groups after CCL4 injection (***P < 0.001); **D** And, 8 weeks after injection of CCL4, detected the expression of SYK by qPCR in siCtrl and siSYK groups (**P < 0.01); **E** Fibrotic liver was detected by photos, HE, masson, Sirius red staining and immunohistochemistry of α-SMA; **F** Determined the expression of TGF-β protein in the liver by Western blot; **G** The expression of α-SMA and Col1A1 in the liver by qPCR; **H** Immunofluorescence of α-SMA to detect the expression in liver tissue (magnification 100x).

So far, a variety of mouse-based experimental models have revealed the intricate roles and mechanisms of SYK in liver diseases [8, 16]. However, it is still unclear whether SYK in monocyte-derived macrophages plays a role in regulating liver fibrosis. Therefore, in order to clarify the role of MoMF SYK in the progression of fibrosis, we continuously and intermittently injected siSYK mixed with mannose-conjugated polymer (JetPEI-polyplus) in mice injected with CCL4 to knock down SYK expression in liver MoMFs. It is well known that the liver has a resident subgroup of macrophages—Kupffer cells. Most of the time during the early stage of liver response to inflammatory stimulation, macrophages in the liver are mixed clusters of Kupffer cells and MoMFs. In particular, it should be noted that we chose to inject siSYK polymer into the tail vein 4 h before each CCL4 injection [17, 18]. The expression of liver SYK 24 h after CCL4 injection was significantly lower than that in the control group (Fig. 2A). Therefore, we focused on exploring which subset of macrophages siSYK target on (Kupffer cells or MoMF). We found that regardless of whether Clodronate liposome (CL) is used to clear KC in advance, the administration of mannose-siSYK will reduce the expression of liver SYK 24 h after CCL4 injection, and the clearance of KC does not cause a significant difference in the level of SYK (Fig. 2B, C). In our animal model, siSYK-mannose conjugated polymer targets MoMFs, rather than resident macrophages in the liver. This indicates that in the damaged liver, SYK responds to the inflammatory environment of the liver and increases dramatically in MoMF. In the model we adopted of long-term injection of CCL4, the expression of SYK mRNA was also decreased in siSYK group (Fig. 2D). From the histological level, siSYK-mannose conjugated polymer can significantly alleviate the degree of liver fibrosis damage. (Fig. 2E) The suppression of TGF-β protein expression also suggests the utility of siSYK (Fig. 2F). α-SMA and Col1A1 reflecting the induction of fibrotic collagen deposition significantly down-regulated in the liver of siSYK (Fig. 2G). Furthermore, a small amount of α-SMA expression (Fig. 2H) was detected by confocal immunofluorescence in the liver of siSYK. In general, the effect of strictly procedurally and continuously administered siSYK-mannose polymer targeting MoMF to inhibit the progression of fibrosis is clear and significant in CCL4-injected mice.

### Inhibition of MoMF SYK blocks Erk phosphorylation

**Fig. 3:**
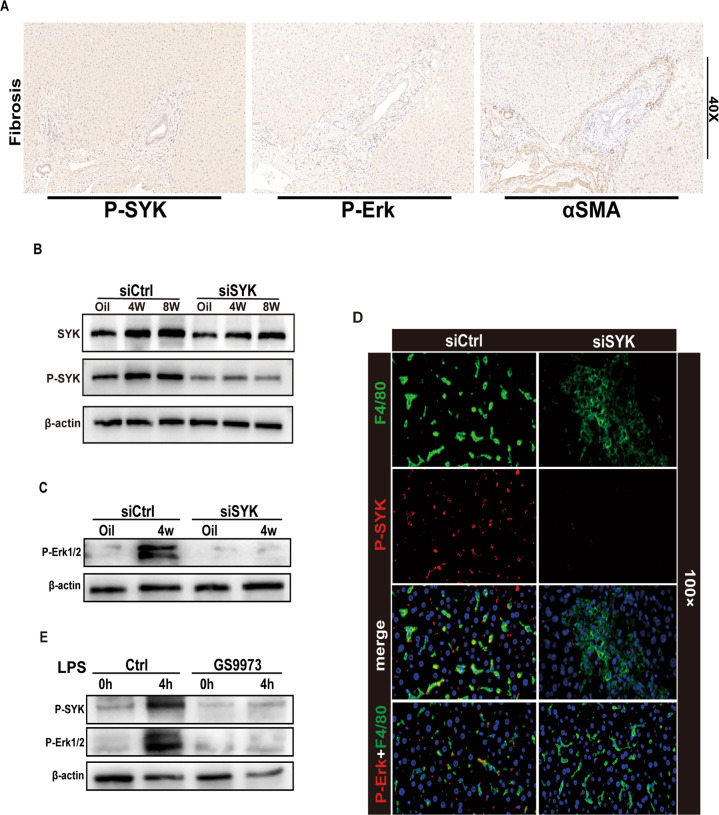
Inhibiting MoMFs SYK blocked Erk phosphorylation. **A** IHC of Human fibrotic liver α-SMA, p-SYK, P-Erk (magnification 40x). **B** Western blot was used to measure the protein expression of SYK and p-SYK in the liver of mice injected with CCL4 4 and 8 w, oil as a control. **C** Detected the protein level of macrophage p-Erk after silencing liver macrophage syk. **D** Confocal immunofluorescence to check the expression of p-SYK and p-Erk in F4/80 macrophages (Zoom 100x). **E** In the presence or absence of the syk inhibitor GS9973, the protein levels of p-SYK and p-Erk were detected after stimulating BMDM with LPS for 4 h.

Although SYK expresses both in liver parenchymal cells and non-parenchymal cells, previous studies have shown that SYK mainly expresses in hepatocytes and HSC in the fibrotic liver [8]. p-SYK plays a regulatory role during the inflammatory injury period in response to stimulation such as ITAM [4, 19], so we speculate that p-SYK plays a vital role in the progression of liver fibrosis. Activation of Erk is important for the progression of liver fibrosis. In the human fibrotic liver, compared with normal liver, the expression of p-Erk increases and has a correlation with the expression of αSMA (Fig. 3A). In fact, the expression of phosphorylated SYK was decreased in response to siSYK. This may be due to the decrease of total SYK protein pool induced by the RNAi of SYK, which leads to the decrease of SYK protein pool that can be phosphorylated [16] (Fig. 3B). In order to explore whether MoMFs p-SYK works through the p-Erk pathway to drive the fibrosis process, we first blocked SYK and detected the expression of p-Erk in macrophages. Inhibition of CD206 + MoMF SYK significantly affect the activation of p-Erk in macrophages (Fig. 3C, D). The results of in vitro stimulation of BMDM by LPS also indicated that blocking SYK activation inhibited the phosphorylation of Erk (Fig. 3E). In general, in the induction of liver fibrosis, activated SYK may play a regulatory role through phosphorylation of Erk.

### SYK relies on phosphorylation of Erk to regulate Hif1α transcription

**Fig. 4:**
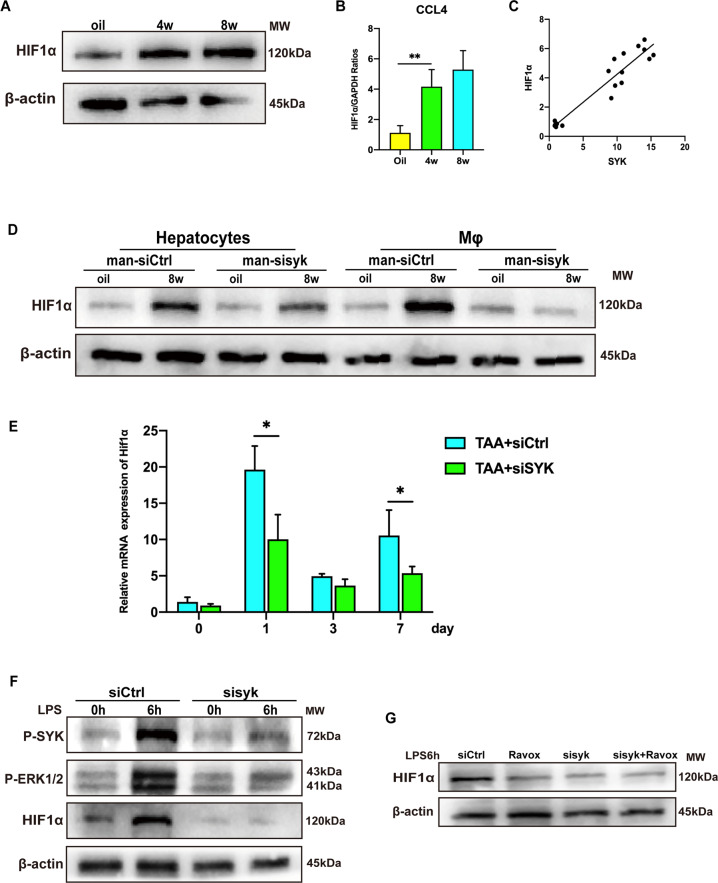
SYK regulates Hif1α transcription depending on Erk phosphorylation. HIF1α **A** protein and **B** mRNA levels were measured after CCL4 injection for 4 weeks and 8 weeks by Western Blot or PCR; **C** The correlation analysis of the expressions of SYK and HIF1α in CCL4 models (**p < 0.01); **D** The hepatocytes and macrophages expression of Hif1α in siCtrl and siSYK fibrotic livers. **E** And, 4 h before TAA injection, mice were injected with siCtrl and siSYK through the tail vein, and determine the transcription of Hif1α in the liver 1, 3, and 7 days after TAA injection Level (*p < 0.05); **F** Transfect mouse BMDM with shSYK lentivirus, then stimulate BMDM with LPS for 6 h, detect the protein levels of p-SYK, p-Erk1/2, HIF1α in macrophages; **G** The protein level of HIF1α in BMDM in the presence or absence of siSYK or Erk1/2 inhibitor Ravox.

Myeloid HIF1α has been shown to promote the progress of liver fibrosis [11–13]. The phosphorylation of Erk in the liver causes the hypoxic environment of the cell, which stabilizes and activates HIF1α in the nucleus. In CCL4 induced fibrotic liver, the expression of HIF1α was increased (Fig. 4A). The transcription of HIF1α is activated and is positively correlated with the transcription level of SYK in mice injection of CCL4 (Fig. 4B, C). What’s more, the expression of HIF1α in macrophages but not in hepatocytes is strongly down-regulated in the fibrotic liver in response to siSYK-mannose polymer (Fig. 4D). In the TAA model [20], the administration of siSYK was confirmed to affect the transcription of Hif1α (Fig. 4E). Knockdown of SYK in BMDM in vitro caused a decrease in the expression of p-SYK,p-Erk and HIF1α (Fig. 4F). Ravoxertinib (MCE, HY-15947) is an inhibitor of Erk kinase, which can effectively inhibit the phosphorylation of Erk1/2. We found that blocking the phosphorylation of Erk in vitro down-regulated the expression of HIF1α. While blocking SYK and Erk at the same time, compared to blocking SYK or Erk alone, the expression of HIF1α was not further down-regulated (Fig. 4G). These data show that the SYK in monocyte-derived macrophages depends on the phosphorylation of Erk to regulate HIF1α, thereby promoting the progression of liver fibrosis.

### Blocking MoMF SYK accelerates the cross-dress of the pro-inflammatory phenotype of monocyte-derived macrophages and reduces the production of CXCL1

**Fig. 5:**
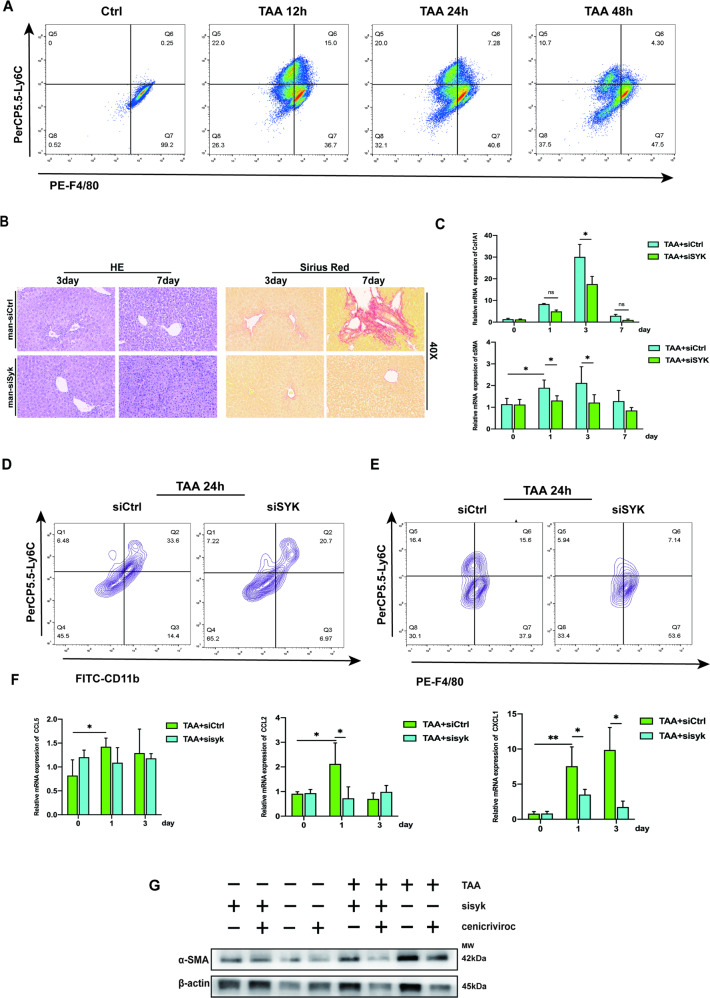
Blocking MoMFs SYK reduced the recruitments of Ly6C^hi^ macrophages and the production of CXCL1. **A** 12 h, 24 h, 48 h after the subacute lethal dose of TAA in mice, F4/80 and Ly6C expression were used to define the phenotypic transition pattern of macrophages. **B** On the 3rd and 7th day after TAA injection, HE and Sirius Red were induced to detect liver fibrosis. **C** mRNA levels of Col1A1, αSMA, in the liver at 1, 3, and 7 days after TAA injection; **D**, **E** Mice were injected with siCtrl or siSYK via tail vein 4 h before TAA injection. Flow cytometry analysis of the expression of CD11b, Ly6C and F4/80 in liver nonparenchymal cells. **F** mRNA levels of CCL5, CCL2, and CXCL1 in the liver at 1 and 3 days after TAA injection; **G** 24 h after TAA injection, the protein level of αSMA in different treatment groups was detected.

It was determined that Ly6Chi monocytes promoted the development of liver fibrosis dependent on CCR2 [21]. Monocyte-derived CCR2 is an important commander in promoting liver fibrosis [22]. CXCL1 has been shown to drive HSC activation and liver fibrosis [23]. IL-1α deficiency can reduce the risk of liver fibrosis in NASH by reducing the level of CXCL1 [24]. At the 12th hour after TAA (100 mg/kg) injection, Ly6ChiF4/80int macrophages had a higher distribution (Fig. 5A), indicating that the inflammatory monocytes at this time had partially transformed into anti-inflammatory resolution macrophages. The recruitment of Ly6ChiF4/80low inflammatory monocytes and the production of Ly6ChiF4/80int transitional macrophages gradually decreased, while the pro-resolution Ly6ClowF4/80hi MoMF which originated from the cross-dress of pro-inflammatory phenotype was gradually increasing. MoMF obtains the expression of CD206 through cross-dress to mediate inflammation repair, which is also the key mechanism for the regulation of siSYK targeting MoMF expressing CD206. In a mouse model with a single sublethal dose of TAA, blocking MoMF SYK can reduce the induction and deposition of collagen fibers (Fig. 5B, C). Moreover, the application of siSYK in vivo reduces the recruitment of Ly6ChiCD11bhi inflammatory cells and Ly6ChiF4/80hi pro-inflammatory macrophages (Fig. 5D, E) and down-regulate the transcription of CXCL1, simultaneously with no significant changes in CCL2 and CCL5 (Fig. 5F). Cenicriviroc is a dual antagonist of CCR2 and CCR5, It has been proven to alleviate liver fibrosis in mice [25], and its anti-fibrotic effect has been demonstrated in the clinical data of phase IIb [26]. In our model, the application of mannose-siSYK did not cause a significant difference in the expression of CCL2 or CCL5, especially during the first 3 days of the fibrosis induction. For this reason, we used Cenicriviroc on the basis of siSYK, and the expression of αSMA was further reduced (Fig. 5G). Therefore, we believe that inhibiting the SYK in monocyte-derived macrophages combined with Cenicriviroc may give patients with liver fibrosis more benefits.

### The risk of high-frequency administration of GS-9973 in the treatment of liver fibrosis

**Fig. 6:**
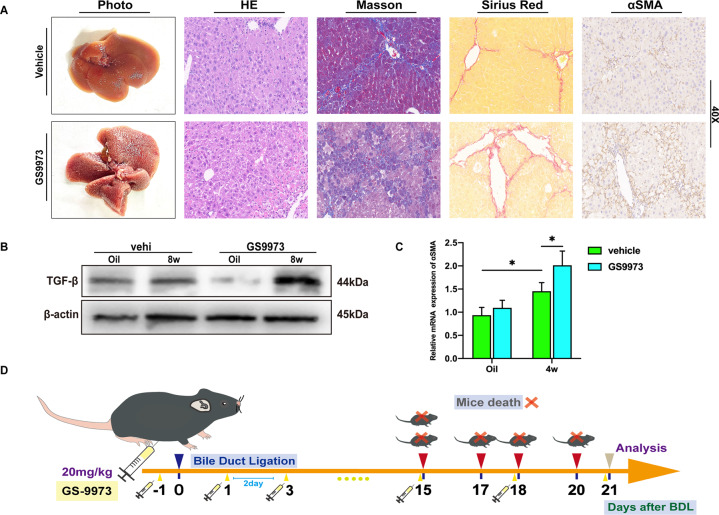
High-frequency injection of GS9973 aggravated liver fibrosis. **A** Mice were intraperitoneally injected with GS9973 (20 mg/kg, q2d), 4 weeks after CCL4 injection, the degree of liver fibrosis was observed by photographs, HE, masson, Sirius red staining, and immunohistochemistry of α-SMA; **B** Mice were injected with GS9973 (20 mg/kg, q3d), the protein level of TGF-β in the liver was measured after 8 weeks of CCL4 injection; **C** The mRNA level of α-SMA was evaluated after 4 weeks; **D** The administration of GS9973 (20 mg/kg, q2d, ip) caused the death of mice with BDL.

As a small molecule inhibitor of SYK, GS9973 has been proven to reduce liver fibrosis in mice [8]. However, continuously intermittent intraperitoneal injection of GS9973 (once every 2 days) caused aggravated liver fibrosis after 4 weeks (Fig. 6A). The expression of α-SMA and TGF-β was also significantly up-regulated compared with the normal saline control group (Fig. 6B, C). Most importantly, in BDL mice, GS9973 caused a strong lethality. All five mice treated with GS9973 died before the end of the assessment. (Fig. 6D) Although the applicability of the dose and frequency of GS9973 is open to discussion, we believe that the clinical trials of GS9973 should be cautious, especially for patients with biliary obstruction.

## Discussion

Liver fibrosis is the pathological repair response of the liver to chronic liver injury. It is a key step in the development of various chronic liver diseases to cirrhosis and an important link that affects the prognosis of chronic liver disease [1, 27]. An acute and chronic liver injury such as hepatitis virus infection, alcohol or non-alcoholic steatohepatitis activates HSC after repeated stimulation, and then HSC gradually differentiates into myofibroblasts, resulting in abnormal distribution and accumulation of hepatic extracellular matrix (collagen, glycoprotein, proteoglycans, etc) [28]. Without active and effective intervention, it can cause structural disorders of liver lobules, nodular regeneration of hepatocytes, formation of pseudo-lobular structures or liver cirrhosis, and manifestations of hypohepatia and portal hypertension, with a significantly increased risk of progression to HCC. [8, 29] About one-third of patients with liver cirrhosis will progress to liver cancer in their lifetime [30]. Correspondingly, most (70–80%) patients with hepatocellular carcinoma caused by hepatitis B have significant liver cirrhosis [31].

SYK orchestrates liver inflammation in response to innate immunity and secondary immunity, and was first found to be highly expressed in hematopoietic cells. Although SYK is widely expressed in parenchymal and non-parenchymal cells of the liver, which has also been verified by us (date not show), two new inhibitors, PRT062607 and Piceatannol, have been studied in myeloid cells to reveal their protective effects on liver fibrosis and liver cancer in vivo. Both inhibitors can selectively prevent SYK phosphorylation, significantly reduce the infiltration of inflammatory cells and inhibit the progression from liver fibrosis to cancer. All the results suggest that myeloid SYK seems to be a very potential target for the treatment of liver disease.

Monocytes that respond to the inflammatory cascade are quickly recruited from the peripheral once the liver is stimulated by DAMPs or PAMPs. In recent years, this theory has been more refined and perfected [32–35]. CD11b + Ly6Chi monocytes recruited to the liver undergo phenotypic transition and then differentiate into macrophages with different functions to finish its’ cross-dressed, thus performing different roles in regulating inflammation [32]. It is certain that the recruitment of monocyte-derived inflammatory cells is one of the important reasons of driving the progression of acute and chronic liver inflammation. Although targeting myeloid SYK appears to be effective in treating liver fibrosis, its precise cellular location and regulatory mechanism are still elusive. Our study highlights the role of SYK in monocyte-derived macrophages, thereby appropriately reclaim a brand new piece of land.

The activation site of Erk is located in the plasma membrane, endoplasmic reticulum and Golgi apparatus. It is the central commander of a series of cell behaviors and plays an important role in regulating cell survival, cell metabolism and cell proliferation [36–38]. Ras-dependent Erk signaling regulates the G1/S cycle transition of proliferating cells [37]. Erk also exerts an apoptosis inhibitory effect in a mitochondrial-dependent manner [36]. The activation of Erk is essential to drive the progression of organ fibrosis. Anti-IL11 treatment blocks the Erk and Smad pathways to inhibit the activation of lung fibroblasts, thereby alleviating lung inflammation and reversing bleomycin-induced pulmonary fibrosis [39]. The application of GHRH-R antagonist MIA-602 alleviates pulmonary fibrosis in mice, and the Erk pathway also plays an important role in this process [40]. In vitro, RNF2 activates the expression of Col1A1 and αSMA in LX-2 cells through the Erk/p38 pathway, which is considered to be a potential target for regulating liver fibrosis [41]. Different cell-targeted Erk pathways may play a heterogeneous role in the progression of fibrosis. In NK cells, mGluR5 regulates the expression of IFN-γ through Mek/Erk, thereby activating the cytotoxicity of NK cells to HSC and driving the resolution of liver fibrosis [42]. ALOX-5 relies on the Erk signal axis to promote the secretion of LTB 4 and LTC 4 to induce HSC activation. Ablation or inhibition of ALOX-5 reduces liver fibrosis [43]. Similar to pulmonary fibrosis, inhibiting IL11 signaling suppresses the phosphorylation of Erk, thereby reducing liver fibrosis in NASH mice [44]. To further understand, IL11RA is highly expressed in human normal hepatocytes and lipotoxic hepatocytes and activates phosphorylation of Erk in response to lipotoxicity, thereby aggravating the progression of NASH [45].

Hif1α has been found to be widely involved in the pathological response to a variety of liver diseases, especially those associated with hypoxia [11, 46, 47]. The morphological imbalance and functional block of the sinusoids of the liver caused by liver fibrosis lead to liver hypoxia and induce the up-regulation of HIF1α expression. At the same time, HIF1a expression changes positively affect the induction of extracellular matrix deposition and angiogenesis in liver fibrosis [48, 49]. Surgical ligation of the bile duct in mice is a common model that induces obstructive cholestatic liver injury and gradually progresses to liver fibrosis. Compared with wild-type mice, HIF1α-deficient mice have a significantly lower risk of liver fibrosis after BDL [12]. Knockout of myeloid HIF1α inhibits the expression of PDGF, α-SMA and Col1A1 induced by BDL, and significantly mitigates the progression of liver fibrosis [13]. In addition, targeting HIF1α in hepatocytes and HSC has also been shown to regulate the occurrence of liver fibrosis in different experimental induction models. Moderate ethanol exposure leads to the activation of HIF1α signal in hepatocytes, induces p53-dependent hepatocyte apoptosis, and exacerbates CCl4-induced liver fibrosis [50]. Loss of HIF1α in hepatic stellate cells will result in the down-regulation of many pro-fibrotic factors such as CCR5 and VEGFα [13]. These studies indicate the driving effect of HIF1α activation in liver fibrosis.

CCR2/CCR5 dual inhibitor Cenicriviroc inhibits the recruitment of Ly6Chi monocytes, thereby improving histological damage and fibrosis in the NASH model [51]. Our research has shown that the combination utilization of siSYK targeting monocyte-derived macrophages and CVC can further inhibit the progression of liver fibrosis on the basis of CVC alone.

Our study highlights the importance of crosstalk of the SYK/Erk/HIF1α axis in MoMFs in driving the progress of fibrosis. What’s more, targeting SYK in MoMFs can enhance the effect of CCR2/CCR5 dual inhibitor Cenicriviroc to inhibit fibrosis. However, our research still has certain limitations. First of all, the superposition of the inflammation cascade in the liver fibrosis model injected with CCL4 for a long period of time leads to a chaotic map of inflammation induction and regression, which to some extent affects our judgment on the targeting of syk, although we determined our treatment is mainly CD206 + MoMF is targeted, but it does not rule out that a small part of kupffer cells that regenerate after death are affected. In addition, we have not been able to further analyze the mechanism by which high-frequency administration of GS9973 leads to exacerbation of death in BDL mice.

In general, targeting SYK in MoMFs seems to be a promising target for liver fibrosis.

### Reporting Summary

Further information on research design is available in the Nature Research Reporting Summary linked to this article.

## Supplementary information

Reporting Summary

## Data Availability

The datasets generated and/or analyzed during the current study are available from the corresponding author on reasonable request.
